# Non-thermal plasma treatment altered gene expression profiling in non-small-cell lung cancer A549 cells

**DOI:** 10.1186/s12864-015-1644-8

**Published:** 2015-06-06

**Authors:** Jue Hou, Jie Ma, K. N. Yu, Wei Li, Cheng Cheng, Lingzhi Bao, Wei Han

**Affiliations:** Center of Medical Physics and Technology, Hefei Institutes of Physical Science, Chinese Academy of Sciences, Hefei, China; School of Life Sciences, University of Science and Technology of China, Hefei, China; Institute of Plasma Physics, Hefei Institutes of Physical Sciences, Chinese Academy of Sciences, Hefei, China; Department of Physics and Materials Science, City University of Hong Kong, Tat Chee Avenue, Kowloon Tong, Hong Kong; Mailbox 1110, 350 Shushanhu Road, Hefei, Anhui 230031 P. R. China

**Keywords:** Non-thermal plasma, Gene profiling, NSCLC A549 cell line, Multiple signal pathways

## Abstract

**Background:**

Recent technological advances in atmospheric plasmas have made the creation of non-thermal atmospheric pressure plasma (NTP) possible for utilization in the medical field. Although accumulated evidence suggests that NTP induces cell death in various cancer cell types thus offering a promising alternative treatment strategy, the mechanism underlying its therapeutic effect is not fully understood.

**Results:**

We analyzed relevant signaling cascades associated with the tumor protein p53, in particular the cell cycle arrest, DNA damage as well as the underlying apoptosis pathways. Based on our results, the major effect from plasma exposure was found to be the activation of MAPK and p53 signaling pathways, resulting in changes in gene expression of MEKK, GADD, FOS and JUN. Finally, a significant modulation in expression of genes related to cellular proliferation and differentiation was observed.

**Conclusion:**

Overall, the presented data of the tumor transcriptome helped identify the key players in modulated gene expression following exposure to plasma at the molecular level, and also helped interpret the downstream processes. The present work laid the foundation for further studies to clarify the roles of multiple pathways in plasma-induced biological processes. Further investigation of these genes in other cell lines may reveal comprehensive mechanisms of plasma induced effects.

**Electronic supplementary material:**

The online version of this article (doi:10.1186/s12864-015-1644-8) contains supplementary material, which is available to authorized users.

## Background

Plasma known as the fourth state of matter is partially or completely ionized gas including a mixture of electrons, ions, radicals, and energetic photons, which is typically generated under high temperature conditions. Due to the interdisciplinary effort of physics and biotechnology, recent technological progress in atmospheric plasmas has led to the creation of cold plasmas with an ion temperature close to the room temperature, which can be controlled or changed according to the desired applications. Moreover, that plasma can interact with organic materials without causing thermal damage to the cell surface, and can effectively inactivate different kinds of pathogens without adversely affecting the healthy tissues. Many other biological applications have also been explored. Accumulated evidence has shown that plasma can be generated and play an increasingly important role in various applications at room temperature, such as blood coagulation, wound healing, and tissue and device sterilization [1]. Furthermore, using endoscopic effect of non-thermal atmospheric pressure plasma has been developed in both clinical applications and disinfection of medical devices [2, 3], as a targeted and low invasive technique. Additionally, some researches have been performed in the utility of cold plasma for anti-tumor or cancer therapy in various tumor cell lines including several solid malignant (cervical carcinoma, lung carcinoma, glioblastoma, thyroid carcinoma, oral carcinoma and colorectal carcinoma) cells [4–6] and lymphoma cells [7, 8]. In particular, it is remarked here that non-thermal atmospheric pressure plasma (NTP) could treat some tumors under the superficial skin such as melanoma, as well as head and neck cancers [9, 10].

Moreover, accumulated evidence has revealed that plasma treatment can induce various effects on multiple cancer cell types, including oxidative stress such as ROS and RNS mediated mitochondria-dependent apoptosis [1, 6, 11], cell cycle arrest, cell growth inhibition [1] and obstructed tumor invasion [12]. The relationships between plasma exposure and cellular responses have been partially established in recent studies which demonstrated that plasma treatment of cells resulted in intense alterations of apoptotic signaling [9, 13] related transcription factors [14], DNA damages [8, 13] and ATM/p53 [9] pathways. In particular, the reactive oxygen/nitrogen species including H_2_O_2_, Ox, NO_2_, NO_x_, which lead to depolarization of the mitochondrial membrane and mitochondrial ROS accumulation that displayed a compromised redox status evident from increased NADP+/NADPH levels, reduced the GSH/GSSG ratios and enhanced cytogenetic damages revealed by micronucleus formation assessment [7, 8]. Moreover, Yan and colleagues elaborated on the several stages involved in the induction of cancer cell death by cold atmospheric-pressure plasma through increased concentrations of NO, ROS and lipid peroxide [15]. In fact, more factors affected the plasma effect on cancer cell viability. Chen defined the “plasma dosage” which characterized the relationship between the characteristics of the cold plasma (including treatment duration, input/output voltage, flow rate and composition of feed gas) and cell viability [4]. In reality, the capability of cell killing by plasma was even found to be sensitive to the concentration of fetal bovine serum in the media as well as the storage temperature for the media [16].

However, the detailed mechanisms underlying these effects have not yet been fully identified. An original research indicated that NTP executed killing effect of human lung cancer cell lines through mitochondrial dysfunction [17]. Moreover, recent studies confirmed these, which reported that NTP induced apoptosis of head and neck cancer cells by a mechanism involving MAPK-dependent mitochondrial ROS [10] and that ROS/RNS triggered JNK and p38 pathways which promoted mitochondrial perturbation and apoptosis [11].

To fundamentally understand and elucidate the effect of NTP on biological pathways, high-throughput microarrays, as an ideal screening tool for gene expression profiling analysis, could help us investigate the underlying genetic characteristic of processes involved in plasma-induced cellular or molecular responses.

The goal of the present study was to analyze the gene expression after NTP exposure that might be involved in cellular apoptosis, signaling transduction and stress response. Therefore, typical non-small cell lung cancer cells A549 were exposed to NTP with helium as the carrier gas. With the high-density microarray technique, the transcriptome for genes regulated by NTP were screened. The results showed different dose-dependent profile patterns upon various NTP exposure durations, and showed that the NTP exposure modulated the transcriptional factors (*e.g.,* Jun, Fos and CEBPB), cytokines (*e.g.,* IL-6, IL-8), leukocyte recruitment and activation factors (*e.g.,* CCL20, CXCL1, CXCL2, CXCL3 and CSF2), enzymes of phosphatase (*e.g.,* DUSP1 and DUSP2) and cyclooxygenase (*e.g.,* PTGS2). Furthermore, several signaling pathways related to stress defensing mechanisms as well as key transcriptional processes were identified.

## Results

### Non-thermal plasma-induced differential gene expression in A549 cell

Using the microarray based approach, we analyzed the cellular gene expression profile of lung adenocarcinoma A549 cells upon treatment with non-thermal plasma, and focused on finding plasma-associated molecular signatures to elucidate the impact of NTP on the transcriptome of this tumor cell.

**Fig. 1:**
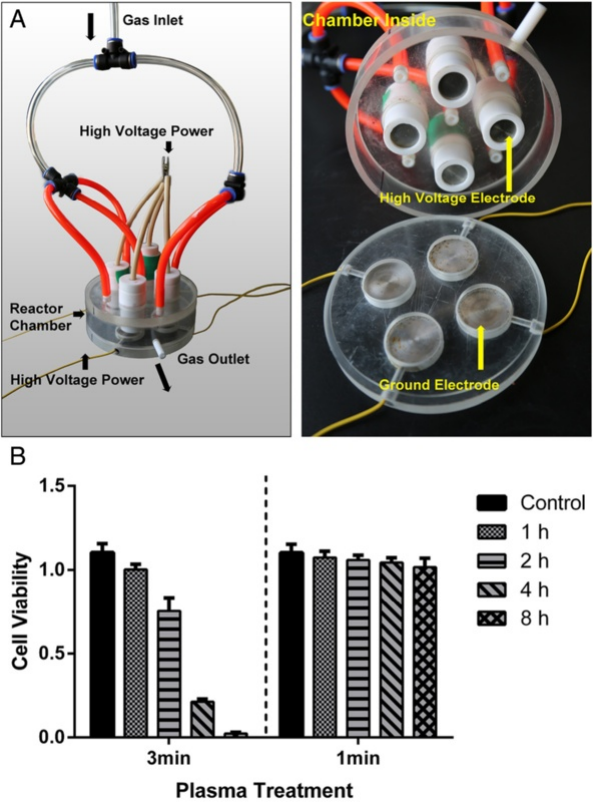
The cell viability assessment of NTP on treated A549. **a** Photographs of a plasma jet and the plasma system. **b** The assessment of cell viability of A549 cell after NTP exposure for 1 min right panel and 3 min left panel, respectively. Time points of 1, 2, 4 or 8 h for viability measurements after NTP exposure were adopted. The mean and SD are shown for three independent experiments. The t-test was applied for the comparative analysis between the treatment group and the control group. The p-values were shown while ns indicated no significant difference

Even though the survival of the 3-min treatment group decreased to only approximate 20 % at 4 h post exposure, when compared to the sham control (Fig. 1), the RNA integrity number (RIN) still showed that RNA was not degraded and had sufficiently high quality for further analysis (data not shown).

**Fig. 2:**
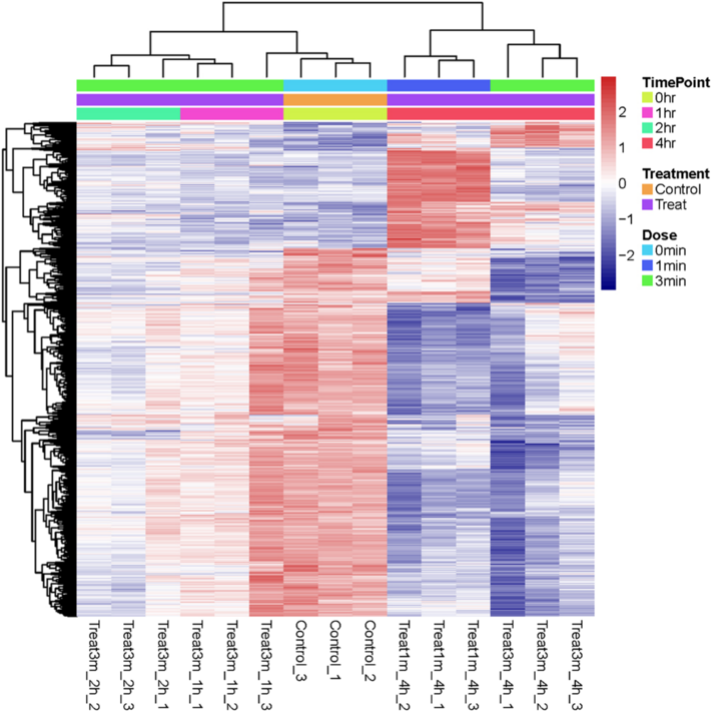
The heatmap of differentially expressed genes. Patterns of changes in transcript abundance are shown on a heatmap for a robust set of 1209 transcripts using a BH *p*-value of 0.05 and fold changes > 1.2. Red color represents relative increase in abundance, blue color represents relative decrease, and white color represents no change. The color bars above the map indicate the time points and NTP doses.

With the selection criteria mentioned above, 1209 differentially expressed genes were obtained for all time points. Specifically, at 4 h after the 1-min NTP treatment, 802 genes (559 down-regulated and 243 genes) showed significant expression according to the preset criteria (with fold changes more than 1.2, and FDR *p* value less than 0.05), whereas only 10 genes (10 down-regulated genes), 132 genes (109 down-regulated and 23 up-regulated genes) and 773 genes (684 down-regulated and 89 up-regulated genes) expressed at 1, 2 and 4 h, respectively, after 3-min NTP exposures (Fig. 2). These data have been deposited in NCBI’s Gene Expression Omnibus and are accessible through GEO Series accession number GSE59997, and the complete overview on the differentially regulated genes could be achieved by GEO2R or other software.

### Gene ontology and pathway enrichment analysis

**Fig. 3:**
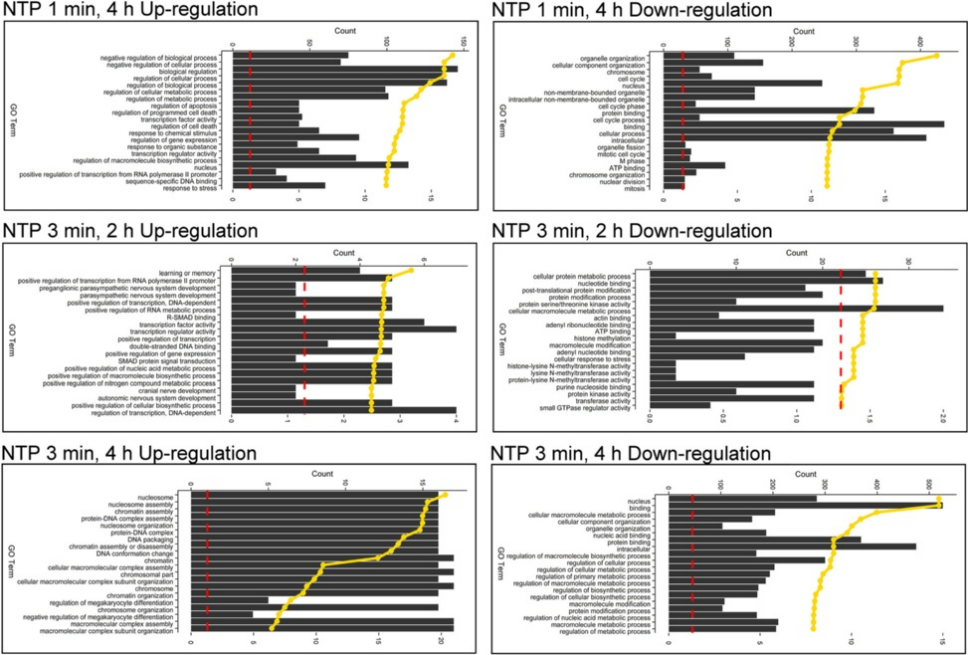
The gene ontology category of each group at indicated time points. The Gene Ontology analysis was performed by BiNGO plugged-in Cytoscape. The Biological Processes (BP), Molecular Function MF and Cellular Components CC terms were involved. To include only significant results, the FDR threshold was set to 0.05. The top 20 terms were selected, and represented as bar plots with gene number involved in these GO terms (with reference to the upper y-axis). Moreover, the corrected *p* values were also obtained as indicated by the yellow dotted line, which had been transformed into log10 with reference to the lower y-axis. The red dashed line indicated the threshold of adjusted p values [−log10 (0.05)].

To retrieve the functional information on differentially expressed genes in each experimental group, co-regulated genes were further classified with BiNGO application with plugged-in Cytoscape. The individual expression products of the identified genes were distributed in different cellular compartments, functional classes, and involved numerous molecular and biological processes. The bar charts (Fig. 3) illustrated the affiliation of the top 20 terms of the regulated genes (FDR *p* < 0.05) with gene class ontologies identified for each group. The whole GO terms for each time point or group could be found in the Additional file 1: Table S1.

Firstly, the GO terms of the whole transcriptome were compared with NTP to understand which categories in response to biological processes were enriched in plasma-treated cells.

At any time point after different NTP treatments, various genes were regulated and involved in a multitude of biological processes. Top hits among the biological process categories also comprised specific aspects of negative regulation of biological process, such as apoptosis and cell proliferation, response to stress and transcription factor activity, which concentrated on up-regulation of gene clusters. Meanwhile, chromosome organization and cell cycle terms were enriched in down-regulated gene clusters at 4 h in the 1-min NTP-exposed group (upper panel in Fig. 3). In contrary to the 1-min NTP exposure, the 3-min NTP treatment caused differential GO categories. At 2 h post the 3-min NTP exposure, the up-regulated genes focused on transcription regulation, and down-regulated genes clustered into nucleotide, metabolic process and methyltransferase activity (middle panel in Fig. 3). Meanwhile, at 4 h after exposure, the top categories comprised nucleosome/chromatin assembly, DNA behavior involved in conformation change and DNA replication in up-regulated gene list, however most of the regulations involved in metabolic processes, nucleus and molecular binding were observed in the down-regulated genes (lower panel in Fig. 3).

**Fig. 4:**
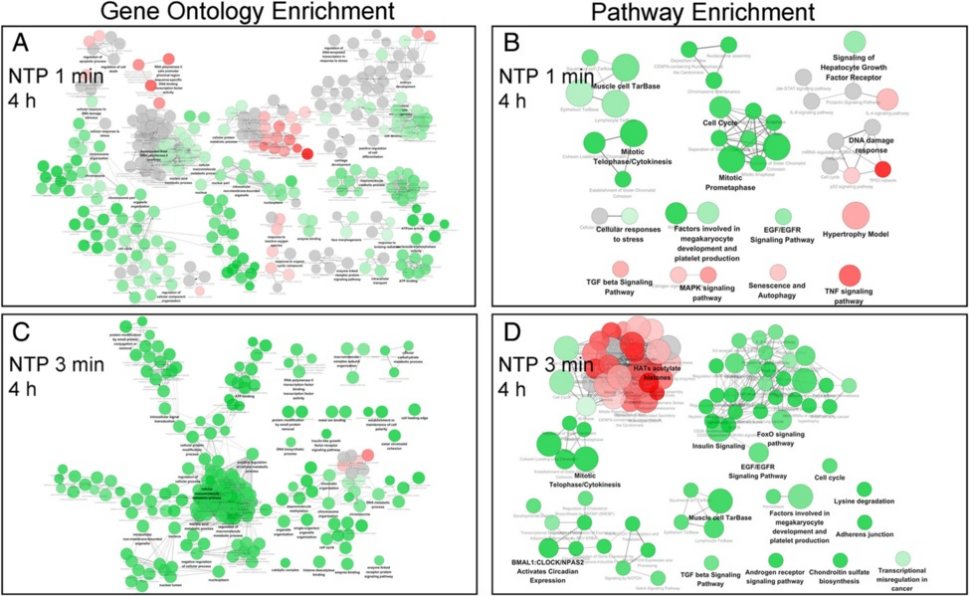
The gene ontology and pathway enrichment results at indicated time points for NTP treatment for 1 min and 3 min. The Biological Processes BP, Molecular Function MF and Cellular Components CC terms were performed for GO enrichment analysis and the KEGG, REACTOME and WikiPathways databases were performed for pathway enrichment analysis of each differential group genes. The statistical test was Enrichment/Depletion two-side hypergeometric test, and the Benjamini and Hochberg false-discovery rate was set to 0.05. Terms with up/down regulated genes are shown in red/green, respectively. The color gradient shows the gene proportion of each cluster associated with the term. Equal proportions of the two clusters are represented in gray. The resulting corrected p values are included in the network visualization by the size of nodes.

In the next step, we utilized gene ontology enrichment analysis to concentrate on the GO categories that were really pivotal at the indicated time point of each group. Briefly, different NTP exposure durations led to distinct time-dependent profiling patterns. Specifically, the down-regulated genes after the 1-min NTP exposure were mainly related to cellular metabolism including chromosome processes, cell cycle, ATPase activity, and nucleoside-triphosphatase activity. Meanwhile, the up-regulated genes were related to kinase activity, transcription factor activity, and response to reactive oxygen species (Fig. 4 A). Interestingly, the gene list for 2 h post 3-min NTP exposure focused on methyltransferase activity, which was a unique gene set among all groups. The enrichment gene sets at 4 h after the 3-min NTP exposure was mostly related to negative or down regulation of cellular processes. These genes were involved in the macromolecule metabolic process, methylation, nucleoplasm, cell cycle, and DNA metabolic process and ATP energy metabolism (Fig. 4 C). All the genes mentioned above are known to be important in the stimulation of cell proliferation and recruitment.

Furthermore, in the pathway enrichment analysis, the regulated genes for 1-min NTP exposure were clustered into biological groups which were different from those corresponding to the 3-min NTP exposure. For instance, it was found that the 1-min NTP exposure significantly repressed the expression of genes involved in mitotic telophase/cytokinesis and prometaphase as well as cell cycle, including DNA damage response and miRNA mediated regulation. Moreover, the 1-min NTP exposure also activated some of pathways including the IL-4 signaling pathway, p53 mediated signaling pathway, MAPK, TGF-β and TNF signaling pathways (Fig. 4 B). However, the 3-min NTP exposure caused up-regulated genes like histones which played a role in HATs acetylate histones and RNA polymerase involved in transcription regulation processes. In contrast, the 1-min NTP exposure reduced the expression of genes involved in regulation of the IL-4 signaling pathway and the TGF-β signaling pathway (Fig. 4 D). However, the effect of extended NTP processing time on gene profiles and cellular behavior and metabolism was not clear.

### Involved hub transcription factors

**Fig. 5:**
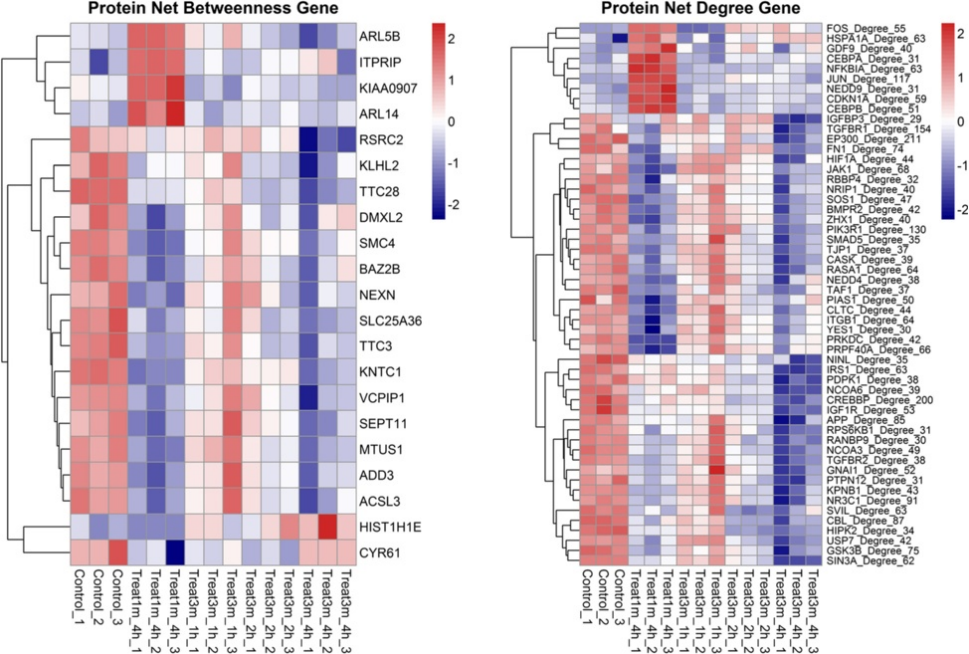
The selected genes from DEG based on protein network betweenness and degree. The genes selected from different expression gene lists, and sorted by top 5 % genes in the protein interaction database according to PID betweenness and degree. Red color represents relative increase in abundance, blue color represents relative decrease, and white represents no change.

Considering that some factors play critical roles in biological processes and interact with abundant related genes or proteins, the top hits proteins were sorted and assessed from protein-protein interaction (PPI) network. Apparently, the 1-min NTP exposure induced specific patterns that included some specifically up-regulated genes. These genes were involved in GTP and GDP energy metabolism (*e.g.,* ARL5B and ARL14), and Ca^2+^ ion flow or release (*e.g.,* ITPRIP) (Left panel of **Fig.**5). Moreover, some transcription factors that played important roles in transcription and regulation were also significantly enhanced (Right panel of Fig. 5). JUN and FOS were thought to play important roles in signal transduction, cell proliferation and differentiation. CCAAT/Enhancer Binding Protein (C/EBP) (*e.g.,* CEBPA, CEBPB) were also important transcriptional activators that regulated expression of genes involved in immune and inflammatory responses. They could bind to regulatory regions of several acute-phase and cytokines genes, and probably played a role in the regulation of acute-phase reaction and inflammation. NFKBIA inhibited the activity of NF-κB/REL complex by trapping REL dimers in the cytoplasm through masking their nuclear localization signals. Additionally, other processes were also present, which involved tyrosine-kinase-based signaling related to cell adhesion (*e.g.,* NEDD9), and cell cycle phase transition (*e.g.,* GDF9).

**Fig. 6:**
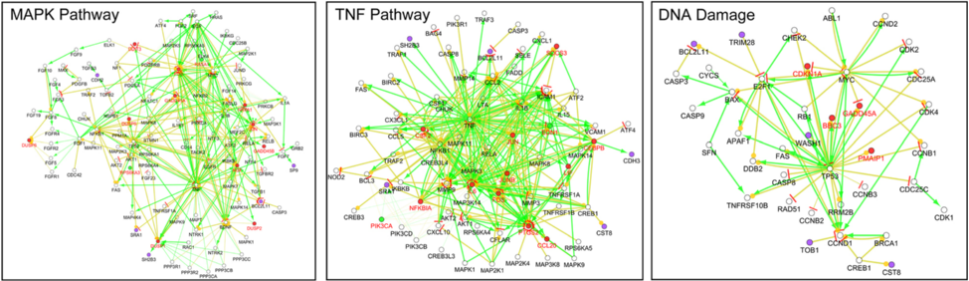
The details of selected pathways after NTP exposure. The illustration showed gene correlation of indicated pathway enrichment in details by CluePedia plugged-in Cytoscape. The nodes represented genes in each pathway, with red and green colors of nodes indicating up- or down-regulated genes, respectively. The green line and yellow line represented activation and expression relationship in gene interaction enrichment, respectively.

### Responded multiple pathways

Differential gene expression in response to the NTP exposure was assessed to elucidate effects of diverse pathways on the cancer cell. Pathway enrichment analysis and PID top hits genes categorized several genes involved in the response to NTP exposure, which comprised transcription factors (*e.g.,* Jun, JunB, Fos and CEBPB), negative intracellular signaling factors (*e.g.,* NFKBIA, SOCS3 and TNFAIP3), leukocyte recruitment and activation factors (*e.g.,* CCL20, CXCL1, CXCL2, CXCL3 and CSF2), enzymes of phosphatase (*e.g.,* DUSP1 and DUSP2) and cyclooxygenase (*e.g.,* PTGS2). In the pathway enrichment results, the up-regulated pathways included IL-4, TNF, TGF-β and MAPK signaling pathways, which constituted a network with centralization of JUN, FOS and JAK critical transcription factors (Fig. 6). Among these, the transcription factors were up-regulated at least 8-fold in the 1-min plasma-treated cells. Next, we focused on changes in expression values of genes whose transcription levels increased under stressful growth arrest conditions. The p53 was a hub of response to stress signals, as shown in the right box of Fig. 6. GADD45 was a DNA-damage inducing gene, mediating the activation of the p38/JUK pathway *via* MTK1/MEKK4 kinase. DUSP also negatively regulated members of MAP kinase superfamily (*e.g.,* MAPK/ERK, SAPK/JNK and p38), which was associated with cellular proliferation and differentiation. All the genes mentioned above displayed enhanced mRNA expression.

**Fig. 7:**
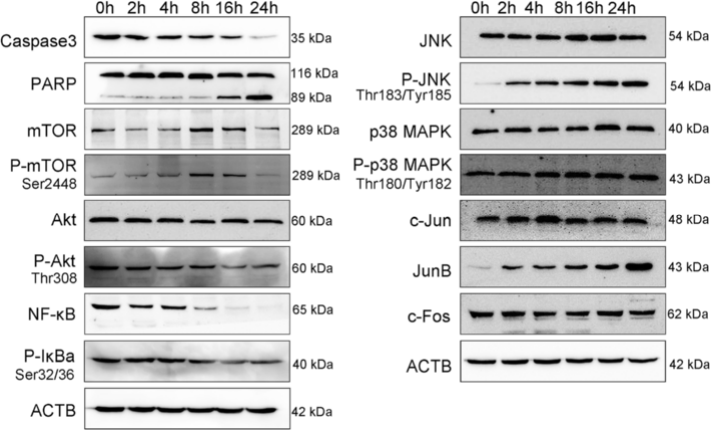
Western blot analysis and validation of several key proteins from microarray results. A549 cells were collected and lysed after 1-min NTP treatment at different time points, namely, 2, 4, 8, 16 and 24 h. With SDS-PAGE gel electrophoresis, the expressions of Caspase3, PARP, mTOR, phospho-mTOR, Akt, phospho-Akt, NF-κB, phospho-IκBα, JNK, phospho-JNK, p38 MAPK, phospho-p38 MAPK, c-Jun, JunB, c-Fos, and ACTB were measured.

### qPCR and Western blot validation

To further validate some of the microarray profiles, seven representative candidate genes were selected for analyzing the gene expression levels *via* the qPCR method. The regulation patterns for all selected genes were consistent with the microarray data and only had marginal differences in the relative fold changes, except the EGR1 and FOS genes which presented extremely high mRNA expression levels by qPCR when compared to microarray analysis (shown in Table 1). Moreover, among the microarray results, we focused on and selected several candidate proteins for multiple pathways, which included apoptosis, MAPK signaling pathway, JNK, Akt and NF-κB pathways, and some critical transcriptional factors (c-Jun, JunB and c-Fos). Among the western blot results, the caspase-3 and PARP degraded over the time points, which indicated the classical apoptosis cascade. mTOR and phosphorylated mTOR levels peaked at 8 h post NTP treatment and descended thereafter. mTOR is a serine/threonine protein kinase which is a central regulator of cellular metabolism, growth and survival in response to hormones, growth factors, nutrients, energy and stress signals [18]. It plays a critical role in the phosphorylation of AKT [19], which has also been validated in our Western blot result in that the expression of phospho-Akt was increased till 8 h after exposure. After that, the Akt regulated the NF-κB signaling pathway by inducing release of NF-κB through phosphorylation and degradation of IκBα (shown in the left panel of Fig. 7). NTP stimuli activated JNK, and phosphorylation of JNK on the site of Thr183/Tyr185 was increased in the time course, which modified the activities of numerous proteins that resided at the mitochondria or acted in the nucleus, and were involved in apoptosis, cell differentiation and proliferation. Moreover, the JNK activated the AP-1 transcriptional factors (c-Jun, JunB and c-Fos) [20]. Only JunB showed continued enhancement with in the time course after NTP treatment, while the expression levels of c-Jun and c-Fos remained stable. Overall, from the translational level results, NTP treatment induced cell apoptosis and cell cycle arrest to assist processes for cell repair.

**Table 1:** Verification of gene expression changes in 1-min NTP Treatment at 4 h after exposure

Gene Symbol	qPCR relative change	Microarray relative change (log_2_ fold change)	p-value
CXCL2	3.115 ± 1.007	1.802 ± 0.029 **	0.0016
CXCL3	2.885 ± 0.296	1.786 ± 0.040 *	0.0364
EGR1	33.712 ± 11.183	5.437 ± 0.129 ***	0.0003
FOS	48.040 ± 18.817	4.650 ± 0.058 ****	<0.0001
JUN	9.808 ± 0.892	3.471 ± 0.003 ****	< 0.0001
JUNB	4.554 ± 0.977	1.945 ± 0.238	0.1118
TNFAIP3	3.194 ± 0.212	2.313 ± 0.019 *	0.0153

Note: The results were shown as mean ± SD in the 2^nd^ and 3ed columns, and asterisks indicated significant differences between two methods found using t-test

## Discussion

The impact of NTP on cancer cells has not been investigated using the microarray approach in detail to date. The present work was the first to employ this high-throughput technique to elucidate the responses of NSCLC tumor cells to a particular NTP treatment *in vitro*. The microarray technique was applied to determine the activities of thousands of genes at once to create a partial picture of cellular functions of A549 cells following NTP exposure. In a previous study [21], the microarray technique had been used and to examine the therapeutic potential of a plasma jet on cancer cell lines and tumors, with a focus on the capability of selective tumor cell eradication and on deregulation of signaling pathways. These selective effects of NTP on different cell types can find important applications in cancer treatment [21].

Besides the apoptotic effects induced by long-time (3 min) NTP exposures to A549 cells, we also focused on the biological effects induced by short-time (1 min) NTP exposures, which were below the levels that induced apoptosis in most of the cells. The time points of 1, 2 and 4 h post cell treatment were selected for detection of early effects in terms of NTP modulated gene activities, which were indeed confirmed by the microarray data.

The expressions of more than 1200 genes were modulated by NTP exposures, with at least 1.5-fold induction or repression when compared to those of non-treated cells. Our results showed that the affected genes differed significantly between the control and treated cells depending on the conditions. Both sub-groups (*e.g.,* up- and down-regulated genes) contained differentially expressed genes encoding for molecules known to participate in the apoptotic process, cellular response to stress, chromosome organization, cell cycle, DNA damage response and miRNA regulation. The signaling and network analysis by ClueGO/CluePedia revealed that certain significantly regulated genes were involved in pathways which led to the p53 signaling pathway, MAPK signaling pathway, TGF and TNF signaling pathways, and IL-4/IL-6 signaling pathways. Likewise, the majority of the biological processes that decreased the expressions were mainly related to chromosome organization/assembly, cell cycle and metabolic processes.

The most striking effect of NTP in the gene expression profiling analysis was the modulation of a large set of genes that were involved in multiple signaling pathways, including PI3K/Akt and NF-κB mediated TNF signaling, MAPK, Jak-STAT, TGF-β and VEGF pathways. To better understand the influence of plasma-induced reactive species and potentially beneficial mechanisms of NTP on A549 cells, we examined the protein expression of apoptosis and related transcriptional factors which were mostly up-regulated or down-regulated after NTP exposure. A number of transcripts were identified to be significantly modulated for transcriptional factors. In this regard, ClueGO predicted the involvement of FOS/JUN/p53 in intracellular signaling in our experimental setup.

Further analysis of our data suggested that NTP might activate a broader range of p53-relevant processes. It is known that p53 is a critical transcription factor which participates in transcription as well as signal transduction, including cell cycle arrest [22], apoptosis [23, 24], DNA repair [25] and damage prevention, cellular senescence and p53 negative feedback. The Western blot assay also confirmed the multiple pathways involved in these complicated processes. The MAPK, JNK and NF-κB pathways were all involved, and played critical roles in the regulation of responses to external stresses.

In summary, the large number of negatively regulated genes involved in biological or cellular processes reflected the cellular responses to NTP, and helped confirm specific pathways or stress responses to NTP. Although the details regarding the activation of specific pathways remained to be elucidated, our data presented new evidence that NTP treatment of A549 cells modulated the transcription of a variety of genes, which might center on p53 and MAPK and TNF signaling pathways, etc. Our data further indicated that it was possible to modulate cellular signaling as well as metabolism and/or apoptosis processes in NTP treated tumor cells on a genetic level.

## Conclusions

Overall, non-thermal plasma treated cells exhibited a pattern typical of damage responses, thereby validating several critical pathways/regulators, presumably mediated through multiplex signaling pathways. Further investigation of these genes in other cell lines may reveal comprehensive mechanisms of plasma induced effects.

## Methods

### Ethics statement

All procedures involving human cell line use were approved by the Hefei Institutes of Physical Science Committee (Chinese Academy of Sciences), where the approval number was 2014021.

### Cell culture

The human lung adenocarcinoma epithelial cell line A549, purchased from ADCC and stored in our laboratory, were cultured in DMEM (Hyclone, Logan, US) supplemented with 10 % fetal bovine serum (FBS) (Hyclone, Logan, US), 1 % penicillin/streptomycin and 2 mM L-glutamine. All cultures were grown in a humidified 5 % CO_2_ atmosphere at 37 °C.

### Non-thermal plasma equipment

The atmospheric pressure dielectric barrier discharge (DBD) plasma is schematically illustrated in Fig. 1 A, which refers to the setup described elsewhere in previous researches [1, 26]. The four DBD plasma reactors are sealed in a hollow plexiglass cylinder as a reactor chamber with two orifices. One is for injection of working gas, and the other is for gas exhaust from the chamber. The high-voltage electrode is a 32-mm-diameter copper cylinder, which is covered by a 1-mm-thick quartz glass as an insulating dielectric barrier. The ground electrode is a 37-mm-diameter copper cylinder. The discharge gap between the bottom of the quartz and the treated sample surface is fixed at 5 mm. The alternating current power supply is a commercial transformer capable of generating continuous and tunable output voltages and frequencies. The applied voltage and discharge current of the DBD plasma are monitored on a Tektronix MSO 5104 digital oscilloscope equipped with a high voltage probe (Tektronix P6015A) and current probe (Tektronix P6021). The gas flow into the chamber from the gas inlets had a fixed flow rate of 80 L/h. In order to expel air as much as possible from the reactor chamber, helium was injected 5 min before the experiment. The non-thermal plasma is generated by a voltage of 12 kV (peak to peak) with a frequency of 24 kHz. The discharge power density was measured to be about 0.9 W/cm3. The typical optical spectrum of the helium DBD plasma was shown in Additional file 2: Figure S1, while the typical current and voltage of the helium DBD discharge were shown in Additional file 3: Figure S2.

### Cell viability assay

The viability of cells was assessed with the Cell Counting Kit-8 (Beyotime, China) following the manufacturer’s instructions. At 24 h after NTP exposure, the cells were treated with CCK-8 reagent for 1 h at 37 °C, and then 200 μl of the supernatant was transferred into the 96-well plates. The absorbance was measured at 450 nm with a Varioskan Flash microplate reader (Thermo Fisher Scientific, Rockford, IL, USA)

### NTP treatment and RNA preparation

A549 cells were passaged 24 h before NTP treatment. Cells (3 × 10^5^) were seeded into 35 mm dishes and were allowed to grow to 80 % confluence before NTP exposure. Cells with 3 ml culture medium were treated directly with NTP. After 1-min or 3-min exposures, the cells were returned into the incubator for further culture for a specific duration, and then harvested by trypsinization and centrifugation. The cell pellets were lysed with TRIzol (Life technologies, Carlsbad, CA, US) and stored at −80 °C for further studies.

The total RNA was extracted with TRIzol Reagent (Life technologies, Carlsbad, CA, US) following the manufacturer’s instructions, and checked for an RNA integrity number (RIN) to inspect the RNA integrity with an Agilent Bioanalyzer 2100 (Agilent technologies, Santa Clara, CA, US).

Qualified total RNA was further purified with RNeasy micro kit (QIAGEN, GmBH, Germany), and genomic contamination was removed with RNase-Free DNase Set (QIAGEN, GmBH, Germany). The purified RNA was stored at −80 °C.

### Microarray hybridization

The total RNA was amplified, labeled and purified using the GeneChip 3’IVT Express Kit (Affymetrix, Santa Clara, CA, US) following the manufacturer’s instructions to obtain biotin-labeled cRNA. After hybridization on Human PrimeView Arrays for 16 h at 45 °C and 60 rpm in Hybridization Oven 640 (Affymetrix, Santa Clara, CA, US), slides were washed and stained with a Fluidics Station 450 (Affymetrix, Santa Clara, CA, US). Scanning was performed on a seventh-generation GeneChip Scanner 3000 (Affymetrix, Santa Clara, CA, US). The Affymetrix GCOS software was used to perform image analysis and to generate raw intensity data. These microarray data have been deposited in NCBI’s Gene Expression Omnibus and are accessible through GEO Series accession number GSE59997.

### Microarray data analysis for Differential Expression Gene (DEG)

All microarray data analyses were performed in R (http://www.r-project.org/) with multiple packages involving the Affymtriex platform for data analysis and statistical analysis in the subsequent Bioconductor. The initial data quality was assessed by the background level, labeling bias, and pair-wise correlation among samples.

For PrimeView Chip, the customized CDF file (version 17, ENTREZG) downloaded from BrainArray website was performed in probe set mapping [27, 28]. IQR was used for raw data filtering with the genefilter package, with the threshold set to remove the intensity less than 20 % of IQR global intensity. Normalization was performed with RMA algorithm which included the global background adjustment and quantile normalization. Empirical Bayes moderation of the standard error and Benjamini and Hochberg false-discovery rate correction for multiple testing were employed, again as implemented in the limma package [29]. Differentially expressed genes were identified with threshold fold changes of more than 1.2 and BH adjusted *p* value less than 0.05.

### Gene ontology categories analysis

To link our data to prior knowledge, we performed GOEA by using the Cytoscape (http://www.cytoscape.org/) plug-in BiNGO (v2.44) [30]. To include only significant results, the FDR threshold was set to 0.05. The bar plot represented gene number and the adjusted *p* value of each gene set were used to visualize the GO categories.

### Pathway enrichment analysis

We performed pathway enrichment analysis by using the Cytoscape plug-in ClueGO (v2.1.1) and CluePedia (v1.1.1)[31, 32]. The KEGG, REACTOME, and WikiPathways database had been used to enrich pathways. The Benjamini and Hochberg false-discovery rate was set to 0.05. For KEGG pathway based data integration and visualization, the pathview package [33] was used to present some specific KEGG pathways in selected and interested pathways.

### Top hits genes of DEG in protein interaction network

HPRD (Release 9) [34] was used to analyze the betweenness centrality and degree of differential expression genes in the network, which had 9,673 nodes and 39,204 PPIs. Among the exclusive experimentally derived protein-protein interaction databases, HPRD was the most complete and well overlapped with other PPI databases [35]. The data were obtained from the GCC (HPRD: 9,270 nodes and 38,855 interactions) by moving the small clusters and single nodes. All the topological parameters were computed based on the GCC [36].After that, the differentially expressed genes were matched to the arranged PPI datasets, and the top hits genes were identified using the characteristics of betweeness centrality and degree in PPI. The selected genes were visualized in a graphic heatmap.

### qRT-PCR analysis of mRNA expression

To validate the differentially expressed mRNAs by real-time PCR, 1 μg of the total RNA was reverse-transcribed with the One Step PrimeScript RT-PCR Kit (TaKaRa, Japan). Quantitative RT-PCR was carried out with a Light Cycler 480 II instrument (Roche, Indianapolis, IN). The primer sequences used for RT-PCR were available on request. Fold changes in the gene expression were calculated with the ΔΔCt method.

### Western blot

In the present experiments, A549 cells were exposed to NTP for 1 min and harvested at 1, 2, 4 and 8 h post exposure. The cells were rinsed once with ice-cold PBS, spun down at 230 g for 5 min and lysed in ice-cold lysis buffer containing protease and phosphatase inhibitors (Thermo Fisher Scientific, Rockford, IL, USA), and then 2 mM phenylmethanesulfonyl fluoride (PMSF) was added. Subsequently, the cells were kept on ice for 30 min and agitated every 10 min to ensure a complete cell lysis. Lysates were centrifuged at 10^4^ g, the supernatants were isolated, and the protein concentrations were adjusted in all samples prior to heating at 95 °C for 5 min in 1× loading buffer (0.25 M Tris, 2 % SDS, 10 % glycerol, 2 % β-mercaptoethanol, 0.004 % bromphenolblue) and then subjected to SDS-PAGE (sodium dodecyl sulfate poly-acrylamide gel electrophoresis) on precast 12 % PAGE gels. Proteins were blotted onto PVDF membranes. Subsequently, unspecific binding was blocked with 0.5 % nonfat milk powder in Tris-buffered saline (20 mM Tris, 13.7 mM NaCl) containing 0.1 % Tween (TBS-T) for 30 min. After this, the membrane was incubated with the corresponding primary antibody Caspase-3(#9665), PARP (#9532), c-Fos (#2250), c-Jun (#9165), JunB (#3753), Phospho-Akt (#2965), Phospho-IκBα (#9246), p38 MAPK (#8690), Phospho-p38 MAPK (#4511), SAPK/JNK (#9258), Phospho-SAPK/JNK (#4668), NF-κB p65 (#4764), mTOR (#2983), Phospho-mTOR (#5536) (Cell Signaling, Beverly, MA, USA) with appropriate dilution at 4 °C overnight. This was followed by washing three times with TBS-T and incubation with horseradish peroxidase-coupled secondary antibodies (1:10^4^) for 1 h at room temperature. After washing three times with TBS-T, the membranes were visualized by using the Western Blotting Substrate system. The same membranes were stripped and relabeled with antibodies directed against the corresponding total protein as well as ACTB as a loading control.

### Statistical analysis

The obtained cell survival data came from three independent experiments. The Western Blot data were typical results. The results were illustrated with Prism 6.0 (GraphPad software, La Jolla, CA, USA). The mean values were plotted as bar charts with error bars, which represented standard deviations. One-way analysis of variances (ANOVA) or t-test was used to determine the statistical significance of the differences between the interested samples and the sham control.

## Availability of supporting data

These microarray data have been deposited in NCBI’s Gene Expression Omnibus and are accessible through GEO Series accession number GSE59997 (http://www.ncbi.nlm.nih.gov/geo/query/acc.cgi?acc=GSE59997).

## Additional files

Additional file 1: Table S1. The Gene Ontology terms of each treatment and time point Additional file 2: Figure S1. The typical optical spectrum of the helium DBD plasma. The optical emission spectra of the helium DBD plasma was recorded by the AvaSpec-2048-8-RM spectrometer with a grating of 2400 grooves/mm. The dominant emission lines illustrated the presence of the helium atom He (447.1, 501.5, 587.5, 667.8, 706.5, and 728.1 nm), OH radical (306–310 nm), and atomic oxygen (656.5 and 777.2 nm,). In addition, the detected reactive species associated with nitrogen are excited nitrogen molecules between 300 and 400 nm. (JPEG 496 kb) Additional file 3: Figure S2. Current (red line) and voltage (black line) diagrams of the helium DBD discharge. The voltages and currents were monitored by a high-voltage probe (Tektronix P6015A) and current probe (Tektronix P6021) via a digital oscilloscope (Tektronix MSO 5104). The voltage (black line) and current (red line) waveforms of helium DBD plasma was acquired and presented. The discharge occurred periodically at a frequency of about 24 kHz, and the input voltage was about 12 kV. The discharge was characterized by multi-current pulse per positive half cycle of the applied voltage, and the current was about 60 mA. The average discharge power obtained according to the ${P}_{\mathrm{ave}}=\frac{1}{T}{\displaystyle \underset{0}{\overset{\mathrm{t}}{\int }}U(t)I(t)dt}$, and was about 29 W. (JPEG 534 kb)

## Abbreviations

BH: Benjamini and Hochberg adjust
CCL: Chemokine (C-C Motif) Ligand
C/EBP: CCAAT/Enhancer Binding Protein
CSF1: Colony Stimulating Factor 1
CXCL: Chemokine (C-X-C Motif) Ligand
DBD: Dielectric Barrier Discharge
DMEM: Dulbecco's Modified Eagle's Medium
DUSP1/2: Dual Specificity Phosphatase1/2
FBS: Fetal Bovine Serum
FDR: False Discovery Rate
FITC: Fluorescein Isothiocyanate
FOS: FBJ Murine Osteosarcoma Viral Oncogene Homolog
GEO: Gene Expression Omnibus
GO: Gene Ontology
GOEA: Gene Ontology Categories Analysis
IL6: Interleukin 6
JNK: c-JUN N-terminal Kinase
JUN: Jun Proto-Oncogene
MAPK: Mitogen-Activated Protein Kinase
NSCLC: Non-Small Cell Lung Cancer
NTP: Nonthermal Plasma
PI: Propidium Iodide
PTGS2: Prostaglandin-Endoperoxide Synthase 2
RIN: RNA Integrity Number
TGF-β: Transforming Growth Factor Beta
