# Supplementary figures and images for: Non-thermal plasma treatment altered gene expression profiling in non-small-cell lung cancer A549 cells
Source: BMC Genomics. 2015 Jun 6;16(1):435. doi: 10.1186/s12864-015-1644-8 (PMC4483225; doi:10.1186/s12864-015-1644-8)

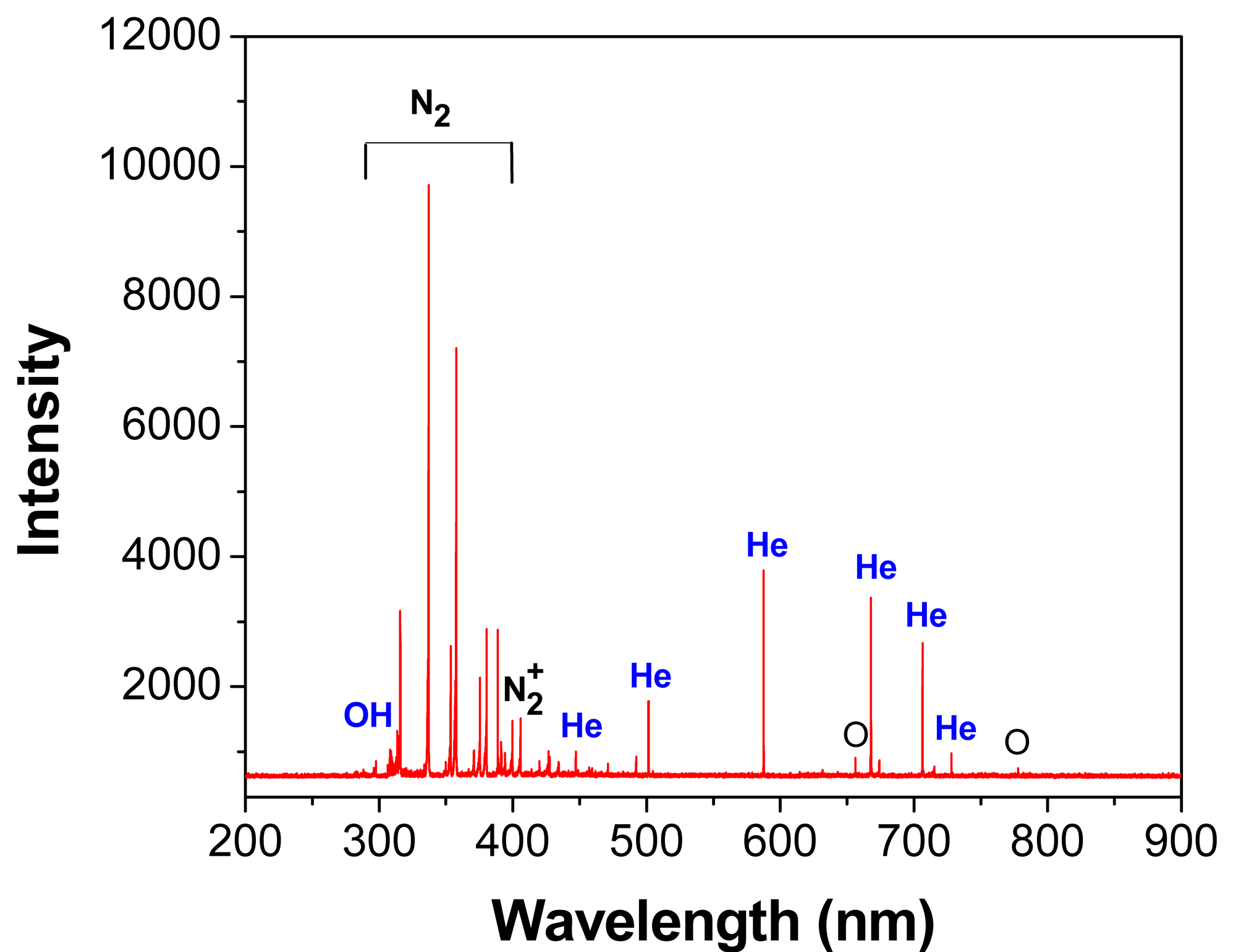

Supplement: Additional file 2: Figure S1. — The typical optical spectrum of the helium DBD plasma. The optical emission spectra of the helium DBD plasma was recorded by the AvaSpec-2048-8-RM spectrometer with a grating of 2400 grooves/mm. The dominant emission lines illustrated the presence of the helium atom He (447.1, 501.5, 587.5, 667.8, 706.5, and 728.1 nm), OH radical (306–310 nm), and atomic oxygen (656.5 and 777.2 nm,). In addition, the detected reactive species associated with nitrogen are excited nitrogen molecules between 300 and 400 nm. (JPEG 496 kb) [file 12864_2015_1644_MOESM2_ESM.jpeg]

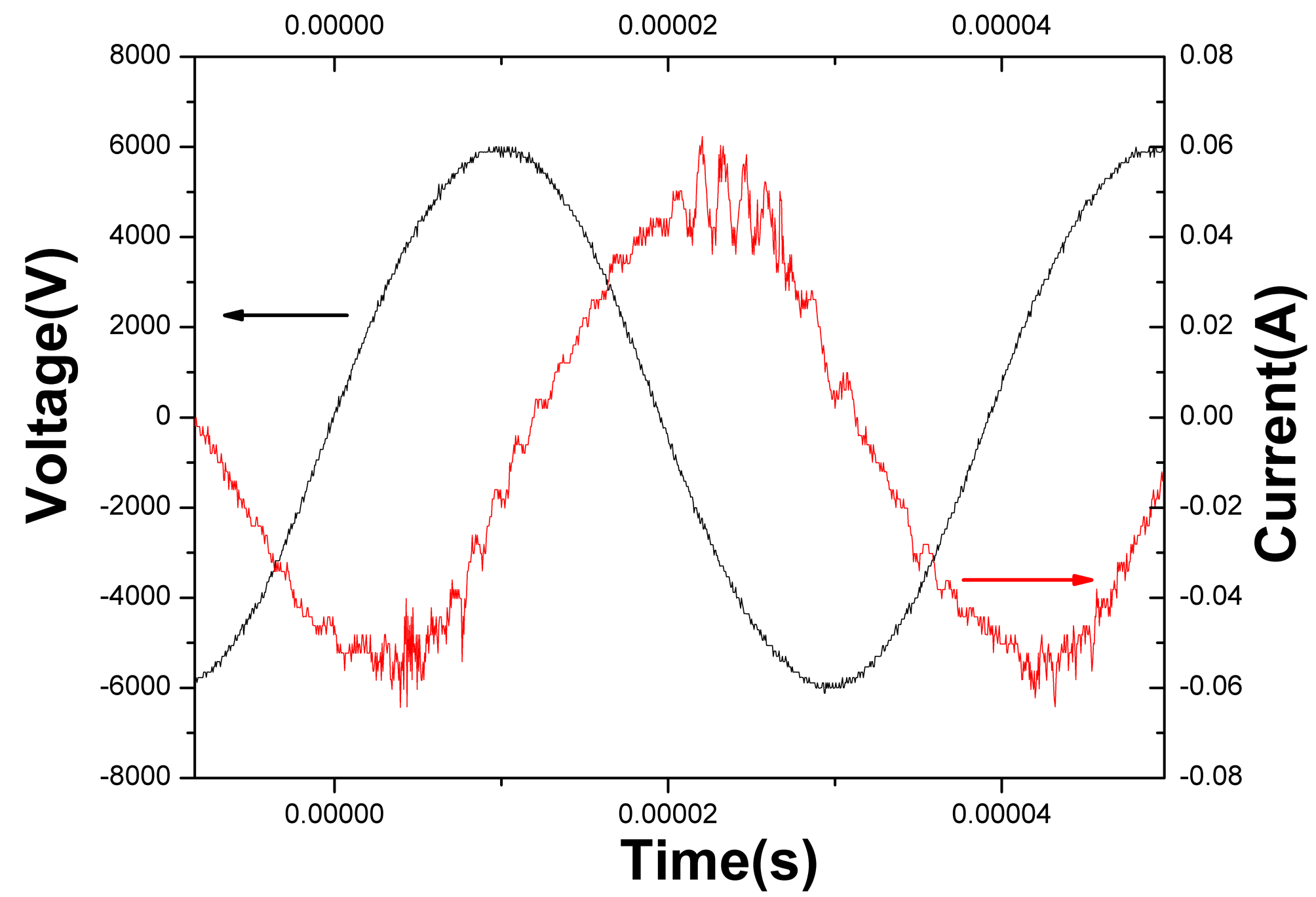

Supplement: Additional file 3: Figure S2. — Current (red line) and voltage (black line) diagrams of the helium DBD discharge. The voltages and currents were monitored by a high-voltage probe (Tektronix P6015A) and current probe (Tektronix P6021) via a digital oscilloscope (Tektronix MSO 5104). The voltage (black line) and current (red line) waveforms of helium DBD plasma was acquired and presented. The discharge occurred periodically at a frequency of about 24 kHz, and the input voltage was about 12 kV. The discharge was characterized by multi-current pulse per positive half cycle of the applied voltage, and the current was about 60 mA. The average discharge power obtained according to the ${P}_{\mathrm{ave}}=\frac{1}{T}{\displaystyle \underset{0}{\overset{\mathrm{t}}{\int }}U(t)I(t)dt}$Pave=1T∫0tUtItdt, and was about 29 W. (JPEG 534 kb) [file 12864_2015_1644_MOESM3_ESM.jpeg]
